# Does Genetic Testing Really Improve the Prediction of Future Type 2 Diabetes?

**DOI:** 10.1371/journal.pmed.0030114

**Published:** 2006-02-28

**Authors:** A. Cecile J. W Janssens, Marta Gwinn, Subramony Subramonia-Iyer, Muin J Khoury

**Affiliations:** **1**Erasmus University Medical Center RotterdamRotterdamNetherlands; **2**Centers for Disease Control and PreventionAtlanta, GeorgiaUnited States of America; **3**Cambridge Genetics Knowledge ParkCambridgeUnited Kingdom

From their study on the genetic prediction of future type 2 diabetes (T2D), Lyssenko and colleagues conclude that “genetic testing might become a future approach to identify individuals at risk of developing T2D” [ 1]. One of their most striking findings is an impressive 21.2-fold increased risk for T2D in obese carriers of the *PPARG* PP and *CAPN10* SNP43/44 GG/TT genotypes with elevated fasting plasma glucose (FPG).

A closer look at their results reveals that the hazard ratio of 21.2 was obtained by comparing the T2D risks of persons who have all three risk factors (“risk genotypes,” obesity, and elevated FPG) with those who have none of these factors. This hazard ratio, thus, measures the combined increase in risk due to *PPARG* PP and *CAPN10* SNP43/44 GG/TT genotypes, obesity, elevated FPG, and their interactions. Among obese persons with elevated FPG, the incidence of T2D was 44.7% in carriers of risk genotypes and 10.7% in persons with other genotypes, yielding a risk ratio of 4.2 (95% confidence interval [CI], 2.3–7.8; follow-up time, age, and sex were not taken into account)—a result that is statistically significant but considerably smaller. Furthermore, genotyping did not significantly alter the risk of T2D in any other subgroups defined by body mass index (BMI) or FPG. Apart from this, the case for predictive genetic testing depends not merely on the magnitude of the risk ratio, but rather on the extent to which the test results are useful for improving prediction of disease [ 2]. In this study, does testing for *PPARG* PP and *CAPN10* SNP43/44 genotypes improve the prediction of T2D based on BMI and FPG alone? The usefulness of genetic testing for predicting disease can be evaluated by comparing the discriminative accuracy of predictions based on models that do and do not include the genetic test results [ 3]. The discriminative accuracy of a test is indicated by its sensitivity and specificity (dichotomous test results), or by the area under the receiver-operating characteristic (ROC) curve (variable test results) [ 4]. Sensitivity is the probability of a positive test result among persons who will develop the disease, and specificity is the probability of a negative test result among persons who will not develop the disease. In a perfect test, both sensitivity and specificity equal one. For tests with variable (not dichotomous) results, a cut-off probability must be chosen. Sensitivity and specificity of the test will vary with the choice of cut-off probability. When sensitivity and specificity are calculated for each possible cut-off value and plotted as an ROC curve, the area under the curve (AUC) measures the discriminative ability of the test. AUC can vary from 0.5 (no discrimination) to one (perfect discrimination) [ 4].

Using the data presented by Lyssenko et al. [ 1], we estimated the discriminative accuracy of predictions of T2D risks that did and did not take genotype into account. We used logistic regression to obtain the risks of T2D for all individuals in the study, considering three different prediction models. In the first model, risk of T2D was predicted by BMI (BMI less than 30 kg/m ^2^; BMI greater than or equal to 30 kg/m ^2^) and FPG (FPG less than 5.6 mmol/l; FPG greater than or equal to 5.6 mmol/l). The second model included BMI, FPG, and carrier status of the combined *PPARG* PP and *CAPN10* SNP43/44 GG/TT genotypes, and the third model also included all interaction effects among the three predictors. ROC curves and estimated values for the AUC were obtained using SPSS 11.0.1. For predictions of T2D risk based on BMI and FPG alone, the AUC was 0.68 (95% CI, 0.63–.073), indicating a moderately discriminative accuracy. In comparison, AUCs of approximately 0.77 have been estimated for both serum cholesterol testing for predicting coronary heart disease and neuropsychological testing for predicting Alzheimer disease in asymptomatic persons [ 5, 6]. When genotype was added to the predictive model including BMI and FPG, AUC remained the same (0.68; 95% CI, 0.63–0.73, for main effect only), even when all interaction effects were considered (0.69; 95% CI, 0.64–0.74). The AUCs of the three prediction models are presented in Figure 1. Thus, the data of Lyssenko et al. suggest that genetic testing for *PPARG* PP and *CAPN10* SNP43/44 genotypes will not improve the prediction of T2D in the presence of information on the nongenetic factors, BMI and FPG. The differences in our conclusions are explained by the fact that Lyssenko et al. focused on the highest-risk category rather than on differences in risk among all participants. Individuals who had 21.2- fold increased risk were already in the highest-risk category based on BMI and FPG alone. Genotyping only further subdivided each risk category, without substantially altering the ranking of the categories. And finally, the 21.2-fold risk applied to the second-smallest subgroup, which included only 38 of the 2,243 participants. Testing for *PPARG* PP and *CAPN10* SNP43/44 genotypes did not change the T2D risk for the vast majority of the population.
